# Platelet-derived growth factor receptor beta activates Abl2 *via* direct binding and phosphorylation

**DOI:** 10.1016/j.jbc.2021.100883

**Published:** 2021-06-16

**Authors:** Kuanlin Wu, Hanzhi Wu, Wanqing Lyu, Youngjoo Kim, Cristina M. Furdui, Karen S. Anderson, Anthony J. Koleske

**Affiliations:** 1 Department of Molecular Biophysics and Biochemistry, Yale University, New Haven, Connecticut, USA; 2 Department of Internal Medicine, Section on Molecular Medicine, Wake Forest School of Medicine, Winston-Salem, North Carolina, USA; 3 Department of Pharmacology, Yale University, New Haven, Connecticut, USA; 4 Department of Neuroscience, Yale University, New Haven, Connecticut, USA

**Keywords:** ABL tyrosine kinase, receptor tyrosine kinase, protein–protein interaction, signal transduction, protein kinase activation, Abl2N, Abl2 N terminus, CD, cytoplasmic domain, DKO, double KO, FA, formic acid, GST, glutathione-*S*-transferase, HA, hemagglutinin, HEK293, human embryonic kidney 293, LSB, Laemmli sample buffer, MBP, maltose-binding protein, PDGFRβ, platelet-derived growth factor receptor beta, SFK, Src family kinase, SH2, Src homology 2, SH3, Src homology 3

## Abstract

Abl family kinases are nonreceptor tyrosine kinases activated by diverse cellular stimuli that regulate cytoskeleton organization, morphogenesis, and adhesion. The catalytic activity of Abl family kinases is tightly regulated in cells by a complex set of intramolecular and intermolecular interactions and post-translational modifications. For example, the platelet-derived growth factor receptor beta (PDGFRβ), important for cell proliferation and chemotaxis, is a potent activator of Abl family kinases. However, the molecular mechanism by which PDGFRβ engages and activates Abl family kinases is not known. We show here that the Abl2 Src homology 2 domain directly binds to phosphotyrosine Y771 in the PDGFRβ cytoplasmic domain. PDGFRβ directly phosphorylates multiple novel sites on the N-terminal half of Abl2, including Y116, Y139, and Y161 within the Src homology 3 domain, and Y299, Y303, and Y310 on the kinase domain. Y116, Y161, Y272, and Y310 are all located at or near the Src homology 3/Src homology 2-kinase linker interface, which helps maintain Abl family kinases in an autoinhibited conformation. We also found that PDGFRβ-mediated phosphorylation of Abl2 *in vitro* activates Abl2 kinase activity, but mutation of these four tyrosines (Y116, Y161, Y272, and Y310) to phenylalanine abrogated PDGFRβ-mediated activation of Abl2. These findings reveal how PDGFRβ engages and phosphorylates Abl2 leading to activation of the kinase, providing a framework to understand how growth factor receptors engage and activate Abl family kinases.

Abl family nonreceptor tyrosine kinases, comprised of Abl1 and Abl2 in vertebrates, translate signals from growth factors and adhesion receptors to regulate cytoskeleton organization and remodeling, which is essential to many cellular processes including cell morphogenesis, adhesion, and migration (1, 2, 3, 4, 5, 6, 7, 8, 9). The catalytic activity of Abl family kinases is important in promoting actin-based cell edge protrusions, facilitating endocytosis and phagocytosis, mediating DNA damage responses, and regulating cell survival and proliferation in a variety of cell contexts. These processes play essential roles in the development and function of the cardiovascular, brain, and immune systems, among others (6, 8, 10, 11, 12, 13, 14, 15, 16, 17, 18, 19).

The catalytic activity of Abl family kinases is tightly regulated, and inappropriate kinase regulation drives leukemia development and promotes solid tumor progression (18, 20, 21, 22, 23, 24). The kinase activities of Abl1 and Abl2 are regulated by a complex set of intermolecular and intramolecular interactions and post-translational modifications (25, 26, 27, 28, 29). Nonactivated Abl kinases are kept inactive *via* an autoinhibitory mechanism, in which the kinase domain is held in a rigid conformation through intramolecular interactions with the Src homology 3 (SH3) and Src homology 2 (SH2) domains (27, 28, 29, 30). Models for kinase activation proposed that engagement of SH3 and SH2 domains with cellular binding partners relieves this inhibition. Subsequent tyrosine phosphorylation events promote adoption of an active conformation and prevent returning back to the inactive conformation (9, 25, 26, 29, 31). Endogenous Abl kinases are activated by diverse stimuli including growth factors, cytokines, DNA damage, and adhesion receptors (1, 2, 3, 4, 7, 9, 31, 32).

Abl family kinases are activated downstream of receptor tyrosine kinases in fibroblast and cancer cells, including the epidermal growth factor receptor and platelet-derived growth factor receptor (PDGFR) (1, 2, 7, 18, 21, 23). The PDGFR beta (PDGFRβ) is an especially potent activator of Abl family kinases, and Abl kinases mediate the biological effects of PDGF including PDGF-induced dorsal membrane ruffles, cell proliferation, and chemotaxis (1, 2, 3, 7, 33). PDGFR signaling through Abl1 is upregulated during the development of resistance to aromatase inhibitor treatment in breast cancer (34). Previous work showed that the PDGFRβ binds Abl kinases, and this is associated with increased Abl kinase activation (1, 2, 3, 7), but the molecular mechanism by which PDGFRβ engages and activates Abl family kinases is not known.

Here, we report the molecular mechanism by which PDGFRβ interacts with, phosphorylates, and activates Abl2 kinase. We found that PDGFRβ binds and phosphorylates Abl2 both *in vitro* and in cells. We also identified several novel tyrosine (Y) phosphorylation sites on Abl2 including Y116, Y139, and Y161 on the SH3 domain and Y299, Y303, and Y310 on the kinase domain. Of notable interest, Y116, Y161, Y272, and Y310 are all located near the SH3/SH2–kinase linker interface, which is crucial for keeping Abl2 in an autoinhibited conformation. Mutation of Y116, Y161, Y272, and Y310 to phenylalanine abrogated PDGFRβ-mediated activation on Abl2. These findings provide a mechanism to understand how Abl family kinases are regulated by receptor tyrosine kinases through different phosphorylation events.

## Results

### The Abl2 SH2 domain binds to phosphotyrosine 771 in PDGFRβ

Previous work demonstrated that Abl2 coimmunoprecipitates with PDGFRβ from cell lysates (3), but whether the PDGFRβ binds Abl2 directly or which protein–protein interfaces mediate this interaction is not known. Upon activation by PDGF binding, the PDGFRβ cytoplasmic domain (CD) undergoes tyrosine autophosphorylation at multiple sites, which recruit key adaptor and signaling proteins. We hypothesized that Abl2 SH2 domain directly binds one or more of these phosphotyrosines.

**Figure 1:**
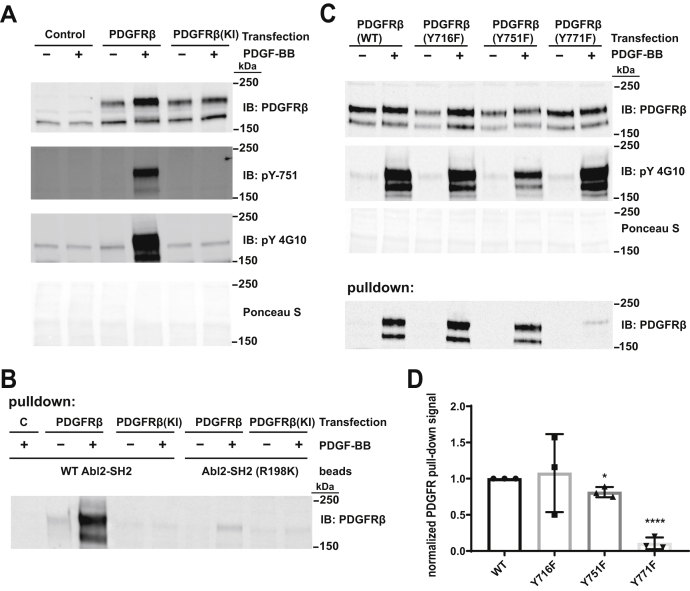
**The Abl2 SH2 domain binds phosphorylated PDGFRβ.***A*, HEK293 cells either untransfected (control) or transfected with PDGFRβ or kinase inactive (KI) PDGFRβ were serum starved overnight and treated with 100 ng/ml PDGF-BB for 10 min. About 40 μg of lysate was immunoblotted with antibodies to PDGFRβ, phosphotyrosine-751 in PDGFRβ, or a general phosphotyrosine antibody (4G10). *Bottom panel* shows one of the Ponceau S-stained blots as loading controls. Molecular weight markers are indicated. Significant tyrosine phosphorylation is shown by Western blotting using both general phosphotyrosine antibody and a site-specific phosphor-Y751 PDGFR antibody. *B*, agarose beads covalently coupled to WT Abl2 SH2 domain and phosphotyrosine-binding defective (R198K) Abl2 SH2 mutant were incubated with 500 μg HEK293 cell lysate expressing WT or KI PDGFRβ that were mock treated or stimulated with PDGF-BB after overnight serum starvation as in (*A*). WT Abl2 SH2 domain pulls down PDGFRβ that had been stimulated with PDGF-BB. *C, top three panels,* WT and tyrosine (Y) to phenylalanine (F) PDGFRβ mutants (Y716F, Y751F, and Y771F) were transiently expressed in HEK293 cells and stimulated as in (*A*). About 40 μg of cell lysate was immunoblotted for PDGFRβ or phosphotyrosine, whereas *bottom panel* shows one of the Ponceau S-stained blots as control. *Bottom panel,* 500 μg of the indicated lysates were incubated with beads coupled to the Abl2 SH2 domain, and the bound material was immunoblotted for PDGFRβ. *D*, quantification of WT and mutant PDGFR pulldown by Abl2 SH2 domain in (*C*). Error bars represent standard error from n = 3 for each condition. ∗*p* < 0.05; ∗∗∗∗*p* < 0.0001. HEK293, human embryonic kidney 293; PDGFRβ, platelet-derived growth factor receptor beta; SH2, Src homology 2.

PDGFRβ was expressed in human embryonic kidney 293 (HEK293) cells and activated by stimulation with PDGF-BB (PDGF). Following stimulation, we found that PDGFRβ could be retained on Abl2 SH2 domain–containing agarose beads but not on beads containing the Abl2 SH2 R198K point mutation that disrupts SH2 binding to phosphotyrosine-containing binding partners (6, 35) (Fig. 1*B*). In parallel, the kinase-inactive PDGFRβ point mutant (K634R) did not undergo PDGF-stimulated autophosphorylation and was not retained on Abl2 SH2 beads (Fig. 1, *A* and *B*). These data indicate that autophosphorylated PDGFRβ in cell lysates can bind the Abl2 SH2 domain.

PDGFRβ is phosphorylated at multiple sites in cells, some or all of which could be binding interfaces for the Abl2 SH2 domain, but previous studies have not resolved which phosphotyrosine (pY) residue(s) of PDGFRβ serve as binding sites for the Abl2 SH2 domain. To address this, we mutated specific tyrosine residues in the PDGFRβ CD to phenylalanine and expressed the mutants in HEK293 cells. All mutants were expressed at similar levels and underwent significant tyrosine phosphorylation following PDGF-BB treatment, but binding of the PDGFRβ Y771F mutant to Abl2 SH2 domain beads was reduced by 90% relative to WT PDGFRβ or any of the other Y to F single-substitution mutants of PDGFRβ (Fig. 1, *C* and *D*). Binding of PDGFRβ Y751F was also reduced but only by 10% relative to WT controls (Fig. 1, *C* and *D*). These data suggest that phospho-Y771 in PDGFRβ is required to interact with the Abl2 SH2 domain.

**Figure 2:**
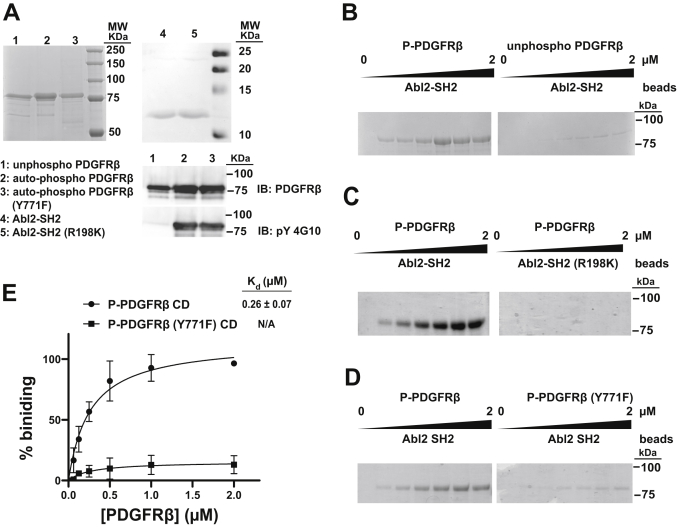
**Abl2 SH2 directly binds phosphorylated PDGFRβ cytoplasmic domain (CD) *in vitro*.***A*, Coomassie *blue*–stained gel showing the purity of recombinant purified proteins used in this figure. About 50 ng of recombinant PDGFRβ CD was immunoblotted with antibodies to PDGFRβ and phosphotyrosine (4G10). *B*–*E*, the concentration dependence of nonphosphorylated PDGFRβ CD (*B*), autophosphorylated PDGFRβ CD (*C*), and autophosphorylated PDGFRβ CD (Y771F) (*D*) binding to Abl2 SH2 domain and phosphotyrosine-binding defective (R198K) Abl2 SH2 mutant (*C*) were measured. An increasing concentration of PDGFRβ CD from 0 to 2 μM in binding reaction was pulled down by agarose beads covalently coupled to Abl2 SH2 at a final concentration of 1 μM. Pulldown products were separated by SDS-PAGE, gel bands were resolved with Coomassie *blue* stain, and densities were quantified using ImageJ. One-site–specific binding isotherms were fit using ImageJ. The *K*_*d*_ value for phosphorylated PDGFR CD and Abl2 SH2 domain is 0.26 ± 0.07 μM. Error bars represent standard error from n = 3. PDGFRβ, platelet-derived growth factor receptor beta; SH2, Src homology 2.

We next used purified recombinant Abl2 SH2 domain and PDGFRβ CD to test whether the proteins interact directly and to measure the affinity and specificity of this interaction. The PDGFRβ CD was comprised of the residues spanning from the C-terminal end of the transmembrane region to the C terminus of the protein (554M-1106L). We purified 6XHis-tagged PDGFRβ CD following baculovirus-mediated expression in insect cells, fully dephosphorylated it *in vitro* using phage lambda phosphatase, and repurified the dephosphorylated PDGFRβ CD (Fig. 2*A*). We incubated PDGFRβ CD in the presence of saturating Mg^2+^ and ATP for 2 h to enable it to autophosphorylate to completion (Fig. 2*A*). Purified autophosphorylated PDGFRβ CD bound to the Abl2 SH2 domain beads with submicromolar affinity (*K*_*d*_ = 0.26 ± 0.07 μM), whereas the nonphosphorylated PDGFRβ CD only exhibited weak background binding (Fig. 2*B*). As in experiments using cell-derived PDGFRβ (Fig. 1*B*), the binding-defective Abl2 SH2 domain R198K mutant completely abolished binding to autophosphorylated PDGFRβ CD (Fig. 2*C*). While the PDGFRβ CD Y771F mutant was able to autophosphorylate *in vitro*, the amount of protein binding to Abl2 SH2 domain–containing beads was greatly reduced compared with WT (Fig. 2, *A*, *D*, and *E*). The Abl2 SH2 domain might bind to other minor phosphorylated tyrosine(s) in the Y771F PDGFR construct, which could explain the small amount of residual PDGFRβ CD Y771F pulled down by SH2 beads (Fig. 2*D*). Together, our data indicate that the Abl2 SH2 domain binds directly to phosphorylated Y771 interface on the PDGFR CD.

### PDGFRβ directly phosphorylates the Abl2 N-terminal half on multiple novel sites

**Figure 3:**
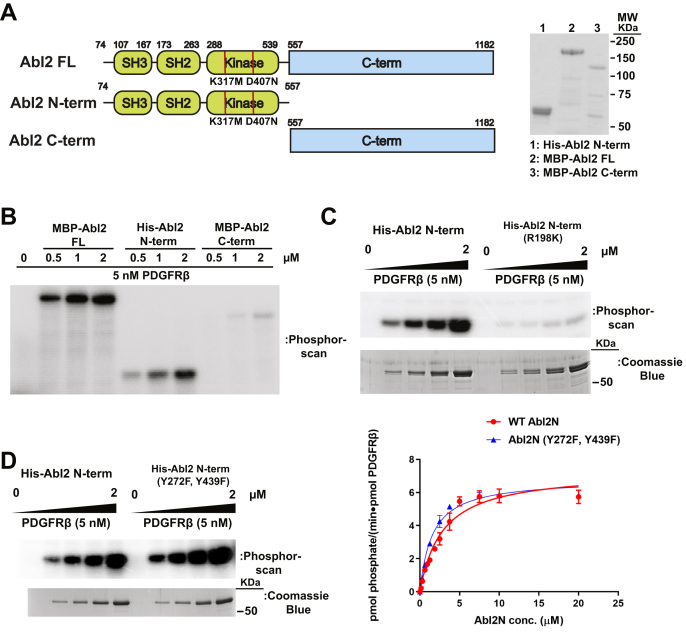
**PDGFRβ phosphorylates the Abl2 N-terminal half.***A*, domain architecture and purified recombinant protein of Abl2 and Abl2 N- and C-terminal halves. Purified proteins were separated by SDS-PAGE and visualized by Coomassie *blue* staining. The Abl2 kinase domain–containing constructs carried two inactivating mutations (D307N, K317M, indicated by *red lines*) in the kinase domain to eliminate possible Abl2 autophosphorylation. *B*, radioactive ATP kinase assays were performed by preincubating 5 nM PDGFRβ CD and 0 to 2 μM Abl2 constructs for 5 min at 32 °C before initiating reactions with 5 μM ATP and 0.75 μCi of [γ-^32^P] ATP for 10 min before terminating with 1× LSB, running on gels, and exposing to a phosphor imaging screen. *C*, radioactive ATP kinase assays testing ability of PDGFRβ CD to phosphorylate Abl2 N-terminal and a phosphotyrosine-binding defective (R198K) Abl2 N-terminal mutant with PDGFRβ CD as kinase. A parallel assay was performed without [γ-^32^P] ATP addition, separated by SDS-PAGE and visualized with Coomassie *blue*. The position of molecular weight was indicated based on gel run under parallel conditions. *D, left panel,* radioactive ATP kinase assays of WT Abl2 N-terminal and Abl2 N-terminal (Y272F and Y439F) mutant phosphorylated by PDGFRβ CD as kinase. A parallel assay was performed without [γ-^32^P] ATP addition, separated by SDS-PAGE and visualized with Coomassie *blue*. The position of molecular weight was indicated based on gel run under parallel conditions. *Right panel,* 0 to 20 μM WT Abl2 N-terminal and mutant were preincubated with 0.1 nM of PDGFRβ CD in kinase assay conditions described in (*B*). Reactions were quenched with 1× LSB after 10 min, boiled, and separated on 10% SDS-PAGE, and protein bands were stained with *Blue* Silver G-250 Coomassie to visualize Abl2 N-terminal protein bands. Bands were cut out, and scintillation was counted. Counts per minute were converted and fit to Michaelis–Menten equation in GraphPad to obtain kinetic parameters. Error bars represent standard error from n = 3. CD, cytoplasmic domain; LSB, Laemmli sample buffer; PDGFRβ, platelet-derived growth factor receptor beta.

Abl2 kinase activity is activated by phosphorylation (26). We used an *in vitro* kinase assay to measure whether purified recombinant PDGFRβ CD phosphorylates Abl2. We expressed maltose-binding protein (MBP)-Abl2 full length (encompassing the first common exon to C terminus), MBP-Abl2 C terminus (residues 557–1182, ∼120 KDa) and a 6XHis-tagged Abl2 N terminus (Abl2N; residues 74–557, ∼55 KDa) in insect cells and purified them (Fig. 3*A*). The Abl2 kinase domain–containing constructs carried two inactivating mutations (D307N and K317M) in the kinase domain, which eliminates possible Abl2 autophosphorylation. In the presence of Mg^2+^ and ATP, 5 nM of recombinant PDGFRβ CD directly phosphorylated full-length Abl2 and the Abl2N but only very weakly the Abl2 C terminus (Fig. 3*B*). We next investigated whether PDGFR/Abl2 direct interaction is required for PDGFR to phosphorylate Abl2. PDGFRβ CD phosphorylation of the Abl2N (R198K) SH2 domain mutant, defective in binding, was greatly reduced relative to WT Abl2N (Fig. 3*C*). These results suggest that SH2 domain–mediated Abl2 recruitment to PDGFR is required for its phosphorylation.

Phosphorylation of Abl2 at Y272 in the SH2 domain-kinase linker and Y439 in the kinase activation loop can activate its kinase activity, and Abl1 is similarly activated *via* phosphorylation of those homologous sites (25, 26). We mutated these sites in the Abl2N construct to test how this impacts phosphorylation by the PDGFRβ CD. Unexpectedly, we found that the PDGFRβ CD could still phosphorylate Abl2N Y272F/Y439F mutant with a similar *k*_cat_ (6.8 *versus* 7.3 min^−1^) and *K*_*M*_ (1.6 *versus* 2.8 μM) as compared with WT, suggesting that the PDGFRβ CD phosphorylates one or more novel sites in Abl2 (Fig. 3*D*).

**Figure 4:**
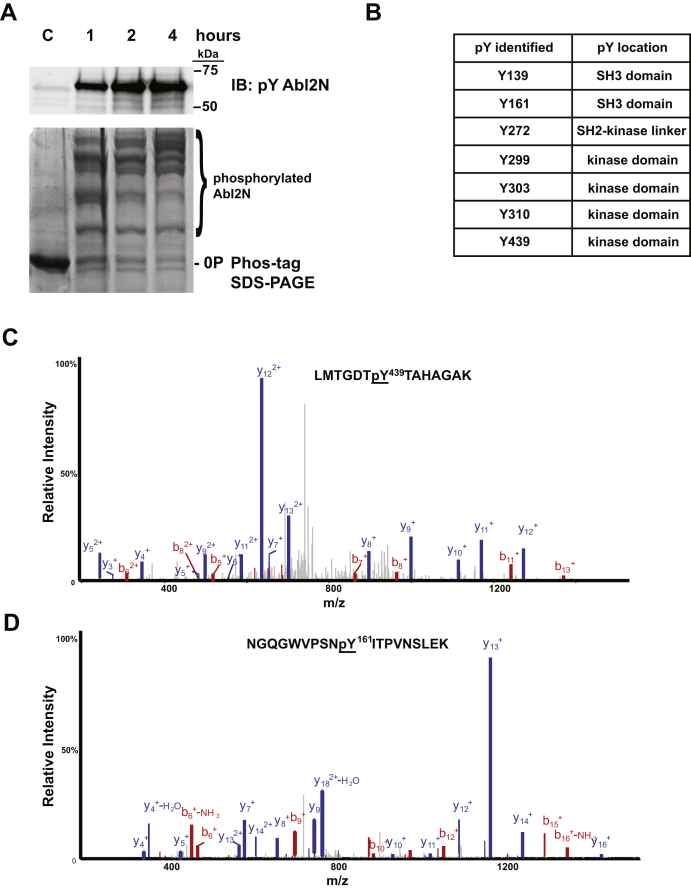
**Phosphopeptide mapping with MS identified novel phosphorylation sites.***A*, phosphorylated Abl2 sample was prepared by incubating 1 μM of Abl2N (KI) with 10 nM of PDGFRβ and Mg^2+^/ATP. Reaction samples were terminated with 4× LSB at 1, 2, and 4 h and monitored for phosphorylation status. *Top panel,* about 100 ng of the reaction sample was immunoblotted with antibodies to phosphotyrosine (4G10). *Bottom panel,* about 10 μg of reaction samples were separated by Phos-tag SDS-PAGE and visualized with Coomassie *blue* stain. *B*, summary of tyrosine phosphorylation sites that are identified by MS with Abl2N phosphorylated by PDGFRβ CD. *C*, the MS/MS spectrum is shown for example of peptides containing Tyr439 with representative b and y fragment ions in *red* and *blue*, respectively. *D*, the MS/MS spectrum of peptides containing Tyr161 with representative b and y fragment ions in *red* and *blue*, respectively. CD, cytoplasmic domain; KI, kinase inactive; LSB, Laemmli sample buffer; PDGFRβ, platelet-derived growth factor receptor beta.

In order to identify the novel phosphorylation sites, we performed phosphopeptide mapping with MS. Abl2N purified from insect cells was treated with a mix of Lambda protein phosphatase and YopH for dephosphorylation. Abl2N phosphorylated by PDGFRβ CD was monitored for phosphorylation status at different time points. Samples immunoblotted for phosphotyrosine show phosphorylation intensity saturates at a reaction time of 2 h (Fig. 4*A*, *top panel*). Phos-tag SDS-PAGE successfully separated unphosphorylated Abl2N and resolved multiple phosphorylated Abl2N species in the PDGFRβ CD-treated samples. As kinase reaction time increases, the intensity increases for higher phosphorylated states of Abl2N, whereas the intensity decreases for lower phosphorylated states and nonphosphorylated Abl2N (Fig. 4*A*, *lower panel*). The phosphorylated Abl2N was used to perform phosphopeptide mapping by MS to locate potential new phosphotyrosine residues. We attempted to limit nonspecific phosphorylation as much as possible by reducing both the kinase concentration and time of phosphorylation. MS analysis identified seven tyrosine phosphorylation sites (Fig. 4*B*), including phospho-Y439, which was previously identified as an Src family kinase (SFK)–mediated phosphorylation site (26) (Fig. 4*C*) and several novel sites including phospho-Y161 (Fig. 4*D*).

**Figure 5:**
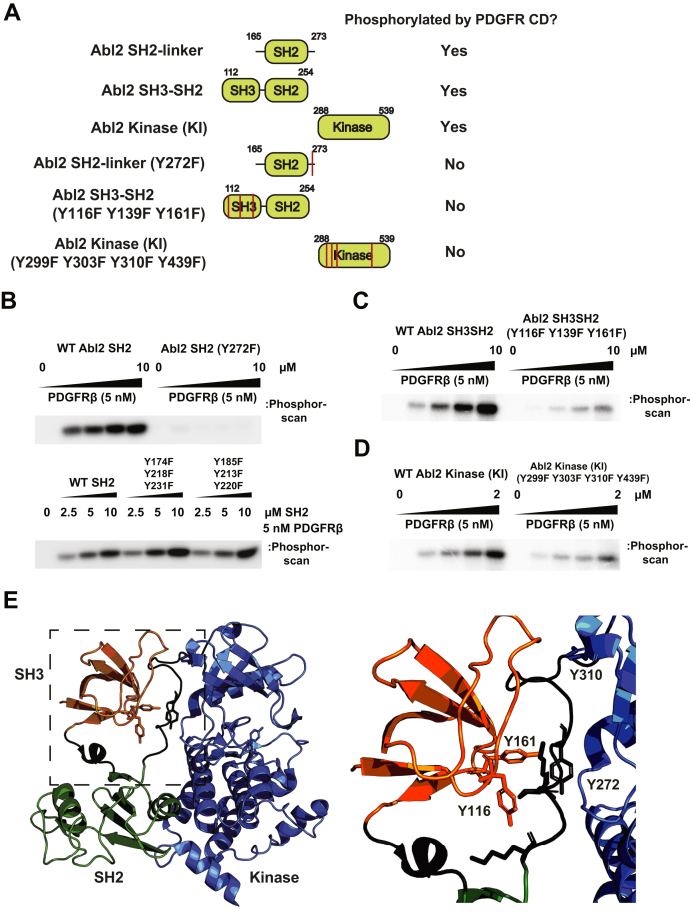
**PDGFRβ phosphorylates Abl2 N-terminal half on several tyrosine residues at the SH3/SH2–kinase linker interface.***A*, domain architecture of the smaller subfragments of Abl2 N-terminal half and their mutant constructs (*red lines* indicate relative point mutation position). The table summarizes whether the construct is phosphorylated by PDGFRβ. *B, top panel,* radioactive ATP kinase assays of WT Abl2 SH2 domain and Abl2 SH2 (Y272F) mutant phosphorylated by PDGFRβ CD as kinase. *Bottom panel*, radioactive ATP kinase assays of WT Abl2 SH2 domain and two SH2 triple mutant constructs. *C*, radioactive ATP kinase assays of WT Abl2 SH3–SH2 domain and Abl2 SH3–SH2 (Y116F, Y139F, and Y161F) mutant phosphorylated by PDGFRβ CD. *D*, radioactive ATP kinase assays of WT Abl2 kinase (KI) domain and Abl2 kinase (KI) (Y299F, Y303F, Y310F, and Y439F) mutant phosphorylated by PDGFRβ CD. *E, left panel,* crystal structure of the autoinhibited state c-Abl1 N terminus (Protein Data Bank code: 2FO0) (28). The position of SH3 domain (*orange*), SH2 domain (*green*), SH2–kinase linker (*black*), and kinase domain (*blue*). *Right panel,* enlargement of the SH3/SH2–kinase linker interface. The SH2–kinase linker adopts a PPII helix that engages the SH3 domain. Abl2 Y116 and Y161 (Y89 and Y134 in Abl1) are located on the binding interface of SH3 domain that faces the linker. Abl2 Y272 (Y245 in Abl1) is located on the linker and faces the N-lobe of the kinase domain. Abl2 Y310 (Y283 in Abl1) is located on the kinase N-lobe and face the linker. CD, cytoplasmic domain; KI, kinase inactive; PDGFRβ, platelet-derived growth factor receptor beta; PPII polyproline type II; SH2, Src homology 2; SH3, Src homology 3.

In parallel with the MS study, we used smaller subfragments of Abl2N as substrates to identify regions phosphorylated by PDGFRβ. We found that the isolated tandem SH3–SH2 domain fragment, a fragment of the SH2 domain containing the SH2–kinase linker, and the kinase domain were all phosphorylated by PDGFRβ, indicating that PDGFRβ can phosphorylate multiple sites as MS study suggests. To cross examine the MS study, we created a panel of Y to F substitution of phosphotyrosine identified by MS and also Y116, which was indicated by MS as a potential phosphorylation site but with lower confidence (Fig. 5*A*). Mutation of tyrosine 272 (Y272F) on the SH2–kinase linker completely abrogated Abl2 SH2 domain phosphorylation by PDGFR, whereas the other triple mutant constructs did not reduce phosphorylation (Fig. 5*B*). Mutation of three tyrosines in the SH3 domain (Y116F, Y139F, and Y161) greatly reduces SH3–SH2 domain phosphorylation by PDGFR (Fig. 5*C*). Mutations of four tyrosines in the kinase domain (Y299F, Y303F, Y310F, and Y439F) also significantly reduced its phosphorylation (Fig. 5*D*). Our mutagenesis kinase assay was consistent with MS findings. Interestingly, there are several novel phosphorylated tyrosine residues (Y116, Y161, Y272, and Y310) located at or near the SH3/SH2–kinase linker interface, which has an important regulatory role of keeping Abl family kinases in an autoinhibited conformation (Fig. 5*E*) (28). We hypothesize that PDGFRβ phosphorylation on these sites would disrupt the autoinhibitory binding interfaces between SH3– and SH2–kinase linker, resulting in Abl2 activation.

### PDGFRβ phosphorylation activates Abl2 kinase activity

**Figure 6:**
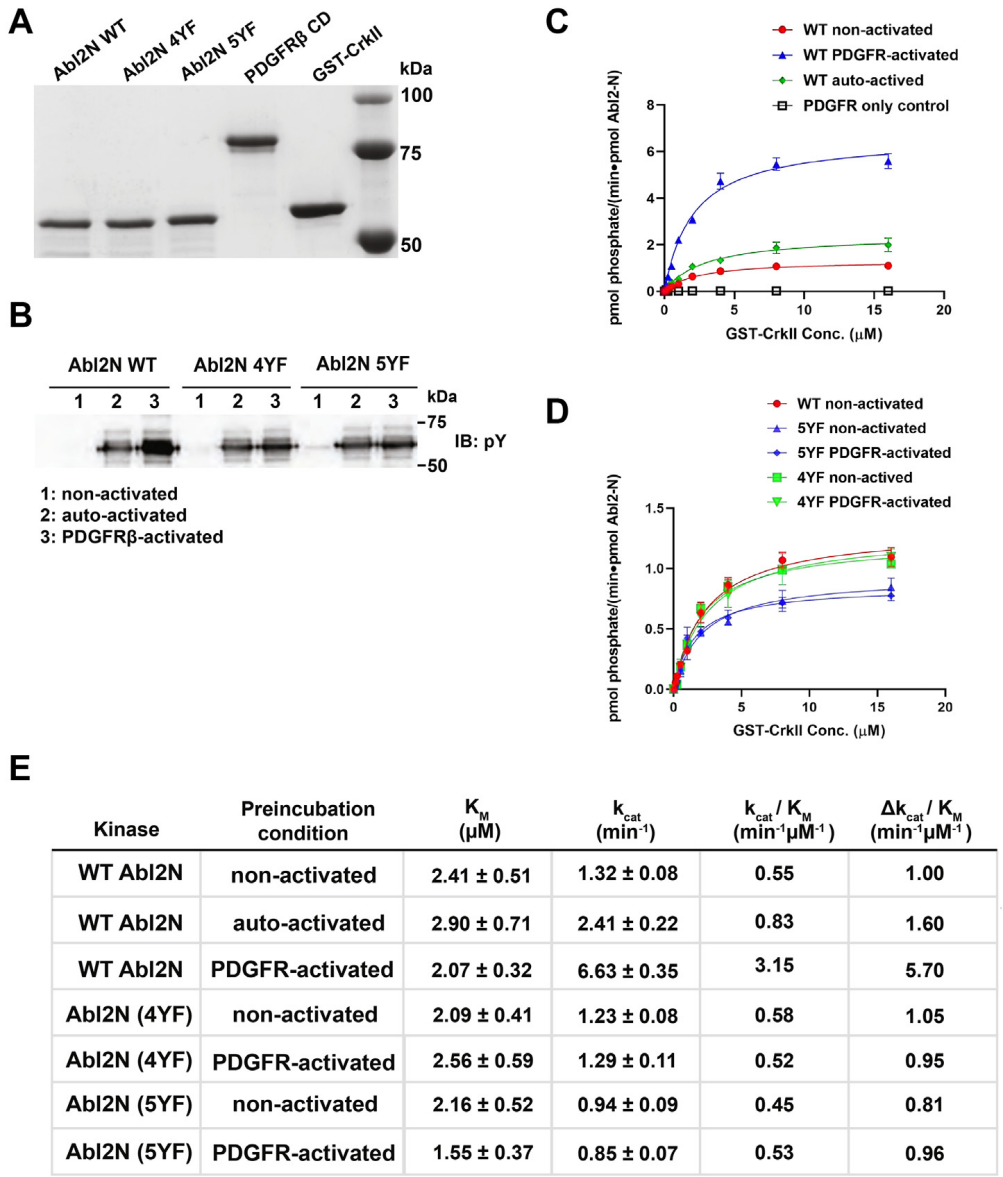
**PDGFRβ phosphorylates Abl2 and modulates Abl2 kinase activation *in vitro.****A*, Coomassie *blue*–stained gel showing the purity of all recombinant purified protein used in this figure. *B*, WT Abl2 N terminus, Abl2-N 4YF (Y116F, Y161F, Y272F, and Y310F), and Abl2-N 5YF (Y116F, Y161F, Y272F, Y310F, and Y439F) were incubated with PDGFRβ in an activation reaction. Control conditions include Abl2-N constructs without PDGFRβ/ATP (nonactivated), and Abl2-N constructs with ATP only (autoactivated). About 100 ng of reaction product were immunoblotted with antibody to phosphotyrosine from all conditions. *C* and *D*, kinase activity was assayed by determining the kinetic parameter of GST-CrkII phosphorylation in [γ-32P] ATP kinase assays. Measurements collected along an increasing concentration (0–16 μM) of CrkII in each condition were fit to Michaelis–Menten isotherms using GraphPad. Error bars represent the standard error from n = 3 concentration series for each condition. *E*, *K*_*M*_, *k*_cat_, and the catalytic efficiency (*k*_cat_/*K*_*M*_) values of Abl2-N mediated GST-CrkII phosphorylation were calculated from isotherms fit to perspective conditions shown in *C* and *D*. GST, glutathione-*S*-transferase; PDGFRβ, platelet-derived growth factor receptor beta.

Autophosphorylation of Y272 in Abl2 and phosphorylation of Y439 by SFKs promotes Abl2 kinase activity (26). We tested if PDGFRβ CD phosphorylation could activate the ability of Abl2 to phosphorylate its substrate CrkII *in vitro*, using purified proteins (Fig. 6*A*). We first incubated 1 μM of Abl2N with 10 nM of PDGFRβ and Mg^2+^/ATP in a 1-h activation reaction, during which we achieved significant tyrosine phosphorylation (Fig. 6*B*). Control preparations include PDGFRβ only, Abl2N without PDGFRβ/ATP (nonactivated), and Abl2N with ATP-only (autoactivated) condition. Following these preincubations, we used 1 nM of Abl2N in kinase reactions to phosphorylate CrkII, and *K*_*M*_ and *k*_cat_ for the reaction were measured. The catalytic efficiency (*k*_cat_/*K*_*M*_) value for the nonactivated Abl2N was determined to be 0.55 μM^−1^ min^−1^, and autophosphorylated Abl2 had a *k*_cat_/*K*_*M*_ = 0.83 μM^−1^ min^−1^, whereas the PDGFR-activated condition was 3.15 μM^−1^ min^−1^. This result suggests that PDGFR phosphorylation on Abl2N promotes a 5.7-fold activation over baseline and a 3.8-fold activation over autophosphorylated Abl2N (Fig. 6, *B* and *D*).

To determine which tyrosine phosphorylation events contribute to Abl2 kinase activation, we selected five tyrosine residues to mutate that were suggested to be most relevant to Abl2 kinase activation (Y116F, Y161F, Y272F, Y310F, and Y439F), by structural modeling, to create the Abl2N 5YF construct. Incubation with the PDGFRβ CD did not result in Abl2N 5YF activation (Fig. 6, *C* and *D*). SFK–mediated phosphorylation of Abl2 on Y439 phosphorylation within its activation loop promotes kinase activation (26). Hence, we also tested whether restoration of Y439, in a 4YF mutant (Y116F, Y161F, Y272F, and Y310F), impacts activation of Abl2N by PDGFRβ. Similar to the effects on the 5YF mutant, PDGFR phosphorylation did not result in Abl2 4YF activation (Fig. 6, *C* and *D*). Using an Abl2 pY439-specific antibody, we found that PDGFRβ does not phosphorylate Tyr439 on the Abl2N 4YF mutant construct, whereas it significantly phosphorylates the WT Abl2N (Fig. S1*A*). We also found that autophosphorylation of the Abl2N 4YF protein does not lead to increased kinase activity (Fig. S1, *B* and *C*). These data suggest that PDGFR phosphorylation on one or more of the additional sites, Y116, Y161, Y272, and Y310, contributes to activation of Abl2N kinase activity.

### PDGFRβ binds and phosphorylates Abl2 in cells

**Figure 7:**
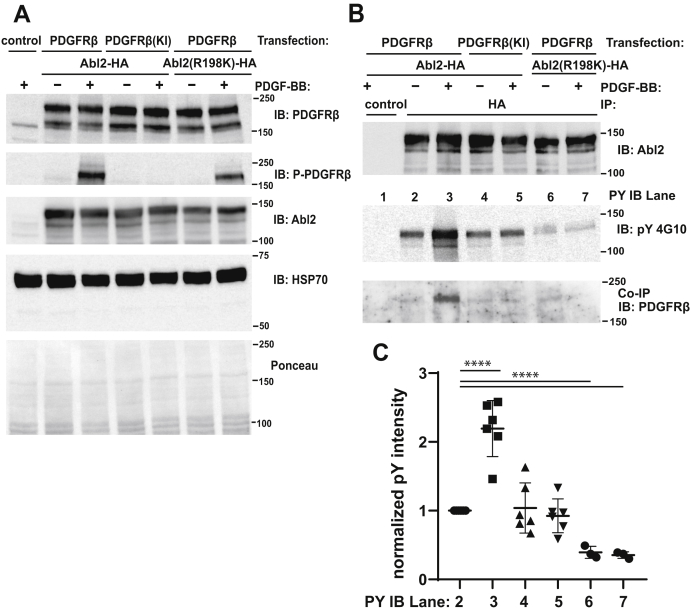
**PDGFRβ forms a complex with Abl2 and phosphorylates Abl2 in cells.***A*, HEK293 cells were either untransfected (control) or transfected with WT or kinase inactive (KI) PDGFRβ and WT or R198K Abl2-HA. HEK293 cells were serum starved overnight and treated with 100 ng/ml PDGF-BB for 10 min. About 40 g of lysate were immunoblotted with antibodies to PDGFRβ, phosphotyrosine, Abl2, or HSP70. *Bottom panel* shows the Ponceau S-stained blot as loading controls. Molecular weight markers are indicated. *B*, about 500 μg of lysate in each condition was immunoprecipitated with anti-HA antibody. Control lane represents lysate pull down by protein-A/G beads without antibody. Immunoprecipitated products were blotted with antibodies to Abl2, phosphotyrosine, and PDGFRβ. *C*, quantification of the normalized phosphotyrosine intensity shown in (*B*) from lane 2 to 7. Error bars represent standard error from n = 3/6. ∗∗∗∗*p* < 0.0001. HEK293, human embryonic kidney 293; PDGFRβ, platelet-derived growth factor receptor beta.

**Figure 8:**
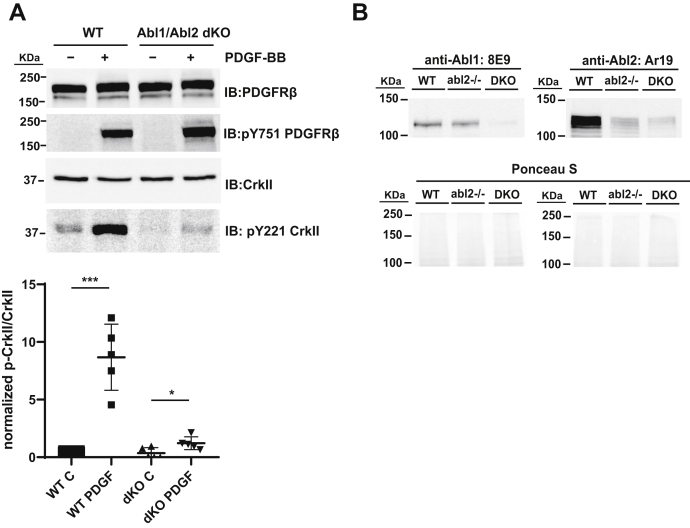
**PDGFRβ signaling promotes Abl2 kinase activation in cells.***A, top panel,* WT and CRISPR Abl1/Abl2 dKO mouse 3T3 fibroblast cells were serum starved overnight and treated with 100 ng/ml PDGF-BB for 10 min. About 40 μg of lysate were immunoblotted with antibodies to PDGFRβ, phosphotyrosine-751 in PDGFRβ, CrkII and phosphotyrosine-221 in CrkII. *Bottom panel,* quantification of normalized p-CrkII/CrkII intensity. Error bars represent standard error from n = 5; ∗*p* < 0.05; ∗∗∗*p* < 0.001. *B, top panel,* WT, CRISPR Abl2 KO, and CRISPR Abl1/Abl2 DKO mouse 3T3 fibroblast cells were immunoblotted with specific antibodies to Abl1 and Abl2. *Bottom panel* shows Ponceau S-stained blot as loading control. DKO, double KO; PDGFRβ, platelet-derived growth factor receptor beta.

We next investigated whether PDGFRβ binds and phosphorylates Abl2 in cells. PDGFRβ and Abl2-HA (hemagglutinin) tag were coexpressed in HEK293 cells, stimulated with PDGF. Both WT and kinase-inactive PDGFRβ and WT and R198K Abl2-HA expressed at similar levels (Fig. 7*A*). Only WT PDGFRβ underwent significant tyrosine phosphorylation following PDGF treatment (Fig. 8*A*). We then immunoprecipitated Abl2 and measured Abl2 tyrosine phosphorylation levels. In cells expressing WT PDGFRβ, PDGF stimulation significantly increased Abl2 tyrosine phosphorylation levels by 2.2-fold, but similar increases in Abl2 tyrosine phosphorylation were not observed in PDGF-stimulated cells expressing kinase-inactive PDGFRβ (Fig. 7, *B* and *C*). Similarly, stimulation of the PDGFRβ did not increase tyrosine phosphorylation of the PDGFRβ-binding defective Abl2 R198K mutant. In fact, the basal tyrosine phosphorylation of the Abl2 R198K mutant was significantly lower than WT Abl2. PDGFRβ also coimmunoprecipitated with Abl2 after PDGF stimulation, but complexes were not detected in transfections expressing either the PDGFR kinase inactive mutant or the R198K Abl2 mutant. These data suggest that PDGFRβ signaling promotes Abl2 tyrosine phosphorylation and PDGFRβ/Abl2 interaction.

### PDGFR activates Abl2 kinase activity in fibroblasts

We next addressed whether PDGFRβ activation leads to Abl2-mediated signaling events in cells. We used CRISPR in WT mouse 3T3 fibroblast cells to achieve 90% reduction in Abl1 levels and 88% reduction in Abl2 levels (Fig. 8*B*). Stimulation of WT mouse fibroblasts with PDGF leads to an eightfold increase in phosphorylation of the Abl1/Abl2 substrate CrkII (Fig. 8*A*), but this was significantly abrogated in Abl1/Abl2 CRISPR double KO (DKO) cells. However, we ascribe the small increase of CrkII phosphorylation after PDGF stimulation to this residual Abl1/Abl2 level in the DKO cells.

## Discussion

We report here the molecular mechanisms by which PDGFRβ binds, phosphorylates, and activates the Abl2 kinase. We provide evidence that Abl2 binds to autophosphorylated PDGFRβ both *in vitro* and in cells *via* the Abl2 SH2/PDGFRβ phospho-Y771 interface. Abl2 recruitment results in PDGFRβ directly phosphorylating the Abl2N on multiple sites. Using both kinase assays with Tyr to Phe substitution and phosphopeptide mapping with MS, we identified up to eight phosphotyrosine sites on Abl2. We demonstrated that PDGFRβ phosphorylation of Abl2 results in Abl2 kinase activation both *in vitro* and in cells. These findings provide a molecular mechanism to understand how receptor tyrosine kinases activate Abl family kinases through different phosphorylation events.

### PDGFRβ may serve as a scaffold to coordinate Abl kinase activation with other signaling outputs

PDGF stimulation induces homodimerization of PDGFRβ as well as heterodimerization of PDGFRα and PDGFRβ, resulting in receptor autophosphorylation at multiple sites. These autophosphorylated Tyr residues serve as docking sites to recruit and activate multiple SH2 domain–containing signaling proteins to elicit specific cellular responses (36, 37). Some of these effectors have intrinsic enzymatic activities, including SFKs, phospholipase C-γ, Ras GTPase–activating protein, and Src homology phosphatase 2 (36, 37, 38, 39, 40). Among them, both SFKs and phospholipase C-γ had previously been shown to activate Abl family kinases through different mechanisms (1, 2, 3, 25, 26). Interestingly, SFKs bind sites on PDGFRβ (pY579/Y581) that are distinct from the pY771 that recruits Abl2. Thus, PDGFRβ may serve as a scaffold to bring these proteins in proximity to promote Abl2 activation. The dimerized form of PDGFRβ may also facilitate Abl2 Y272 autophosphorylation in *trans*, which also promotes kinase activation (25, 26). We anticipate that other cell receptors that activate Abl family kinases, including epidermal growth factor receptor and integrins (4, 9, 21, 41, 42), may similarly use phospho-Y residues to recruit the Abl kinases and coregulators, thereby acting as a platform for Abl family kinase activation.

The identification of phospho-Y771 on PDGFRβ as an Abl2-binding site has implications for additional modes of Abl2 kinase regulation. For example, the Src homology phosphatase 2 tyrosine phosphatase specifically dephosphorylates Y771 in PDGFRβ, which may restrict Abl recruitment to PDGFRβ (43). In addition, Ras GTPase–activating protein also binds to pY771 (39), and it may compete with Abl2 and limit PDGFRβ-mediated Abl2 kinase activation by competing with Abl2 recruitment to the receptor.

### Disruption of the SH3/SH2–kinase linker interaction through phosphorylation may be a common mechanism in abl kinase activation

In the inactive state, the Abl SH3 domain binds to the praline-rich linker between the SH2 and kinase domains, which adopt a polyproline type II helical conformation (28, 44, 45, 46). Mutations of the SH3 domain and the linker prolines perturb this intramolecular interaction, thereby activating Abl kinase activity (30, 47, 48). We found that PDGFRβ phosphorylates Abl2 on four interesting tyrosines (Y116, Y161, Y272, and Y310) that are all located at or near the SH3/SH2–kinase linker interface, which is critical to keep Abl family kinases in an autoinhibited conformation (Fig. 5*D*) (28). Engagement of the Abl2 SH2 domain with PDGFRβ may disrupt this autoinhibited conformation. Subsequent phosphorylation on one or more of these sites would prevent re-engagement of SH3 domain with the SH2–kinase linker and shift Abl2 into a noninhibited “open” activated conformation. Consistent with this, previous studies demonstrated that phosphorylation of Abl2 Y116, Y161, and Y272 (Y89, Y134, and Y245 in Abl1) or mutation of key Pro residues in the SH2–kinase linker prevent engagement of Abl SH3 domain with the SH2–kinase linker and are associated with enhanced Abl kinase activity (26, 48, 49, 50). Abl2 SH3 domain phosphorylation may also result in such open conformation, which releases the SH2–kinase linker. Disrupting the autoinhibited intramolecular interaction within Abl2 could possibly make the linker tyrosine residue (Y245 in Abl1 and Y272 in Abl2) more accessible and more likely to be phosphorylated by either PDGFRβ or by an Abl family kinase in *trans*, which is an important step in Abl kinase activation (25, 26).

### Abl family kinase activation as a multistep process

Phosphorylation of Abl2 at Y439 (Y412 on Abl1) in the kinase activation loop is a critical step for full kinase activation of Abl family kinases (26, 48). However, how this process is regulated is not fully understood. We show that PDGFRβ CD phosphorylation on the Abl2N promotes a 5.7-fold activation over nonactivated Abl2N *in vitro.* Mutation of Y116, Y161, Y272, Y310, and Y439 (5YF) abrogated PDGFRβ-mediated activation of Abl2 kinase activity. Surprisingly, an Abl2N mutant in which Y439 was restored (*e.g.*, the 4YF mutant—Y116F, Y161F, Y272F, and Y310F) could not undergo activation by PDGFRβ. PDGFRβ does not phosphorylate Tyr439 on the Abl2N 4YF mutant construct. Our data may be consistent with a model in which Abl2 Y439 is not efficiently phosphorylated when Abl2 is in a less phosphorylated and possibly more “autoinhibited” conformation. Our model may provide some insight that activation loop phosphorylation and kinase activation may be regulated by N-terminal domain conformation.

Our work adds to a growing body of data indicating that Abl kinases are not simply switched between a closed autoinhibited state and an open active state through a one-step process (9, 25, 26, 29). Instead, Abl kinases appear to be regulated by different types and degrees of intermolecular/intramolecular interactions and post-translational modifications across a spectrum of activity levels (25, 26, 27, 28, 29). Our findings provide a mechanism to understand how Abl kinases are precisely regulated through multistep phosphorylation events by receptor tyrosine kinases.

## Experimental procedures

### Molecular cloning and recombinant protein purification

Full-length Abl2 (residues 74–1182), Abl2 N terminus (residues 74–557), Abl2 kinase domain (residues 288–539), and PDGFRβ CD (residues 554–1106) were cloned with an N-terminal 6XHis tag into the pFastBac1 vector (Invitrogen), as previously described (9). Abl2 C terminus (residues 557–1182) was cloned with an N-terminal MBP tag into the pFastBac1 vector (Invitrogen), as previously described (51). All Abl2 and PDGFRβ point mutants were generated using PCR-based mutagenesis and confirmed by DNA sequencing. Recombinant baculoviruses expressing these constructs were generated using the Bac-to-Bac expression system (Thermo Fisher Scientific) in Sf9 insect cells, as described previously (9). After expression in Hi5 insect cells for 48 h, cells were lysed in Hi5 lysis buffer (50 mM Hepes, pH 7.25, 150 mM NaCl, 5% glycerol, 20 mM imidazole, 1 mM DTT, and protease inhibitors [benzamidine, aprotinin, leupeptin, chymostatin, pepstatin A, and phenylmethylsulfonyl fluoride]). All 6XHis-tagged proteins were affinity purified on nitrilotriacetic acid resin (Qiagen) and eluted with 250 mM imidazole. Proteins were further purified by S200 gel filtration chromatography. MBP-tagged proteins were affinity purified on amylose resin (New England Biolabs), eluted with 10 mM maltose and further purified by S200 gel filtration chromatography. All proteins were buffer exchanged into assay buffer containing 50 mM Hepes at pH 7.25, 150 mM NaCl, 5% glycerol, and 1 mM DTT using 10-ml columns packed with Sephadex G25 resin.

The Abl2 SH3–SH2 and SH2 domains were cloned in frame with glutathione-*S*-transferase (GST) into the pGEX-6P-1 vector, and GST-Abl2-SH3-SH2 and GST-Abl2-SH2 fusion proteins were purified from BL21 (DE3) *Escherichia coli* cells (Millipore Sigma) on glutathione 4B beads (GE Healthcare). The GST tags were cleaved using PreScission protease (GE Healthcare), as previously described (9). GST-CrkII was cloned into pGEX-4T-1 and purified from *E. coli* on glutathione 4B beads (GE Healthcare), as previously described (26). Before use in assays, all proteins were buffer exchanged into assay buffer containing 50 mM Hepes at pH 7.25, 150 mM NaCl, 5% glycerol, and 1 mM DTT using 10-ml columns packed with Sephadex G25 resin.

### Crosslinking of recombinant proteins to beads

AminoLink (Thermo Fisher Scientific) beads were used to covalently link Abl2 SH2 domain following purification (9). Briefly, proteins were gently rotated with AminoLink beads overnight. About 50 mM sodium cyanoborohydride was added to catalyze the reaction. Protein was linked at a final reaction concentration of 1 μM, and the remaining reactive sites on protein-linked beads were blocked with 1 M Tris–HCl, pH 8.0, and 100 mg/ml bovine serum albumin, washed, and stored in assay buffer.

### Binding assays

Binding assays were conducted as previously described (9). For determination of the PDGFRβ CD-Abl2 SH2 domain–binding interface, purified Abl2 SH2 and SH2 (R198K) were covalently linked to AminoLink beads as described previously and added to binding reactions at a final concentration of 1 μM. For determination of *K*_*d*_ values, an increasing concentration gradient of PDGFRβ CD constructs from 0 to 2 μM was used. Binding reactions were incubated for 1 h at 4 °C before washing and resuspending in Laemmli sample buffer (LSB). Bead-associated material were boiled and separated on SDS-PAGE gels. Gel bands were resolved with Coomassie blue silver stain, and densities were quantified using ImageJ (the National Institutes of Health) (52). For measurements of *K*_*d*_, band densities were plotted against concentration of the free solution protein, and binding isotherms were set using GraphPad software using the one-site–specific binding equation, $\;Y\;=\;B_{max}*X/\left(K_{d}\;+\;X\right)$, where *Y* is specific binding, *X* is the concentration of the ligand, *B*_*max*_ is the maximum specific binding, in the same units as *Y*, and *K*_*d*_ is the binding affinity in the same units as X.

### *In vitro* kinase assays

Kinase assays were performed by preincubating 5 nM PDGFRβ CD and 0 to 2 μM Abl2 constructs in 50 mM Hepes at pH 7.25, 150 mM NaCl, 5% glycerol, 5 mM MgCl_2_, 5 mM MnCl_2_, 1 mM sodium pervanadate, and 1 mM DTT for 5 min at 32 °C before initiating reactions with 5 μM ATP with 0.75 μCi of [γ-^32^P] ATP for 10 min before terminating with LSB, running on gels, and exposing to a phosphor-imaging screen. Screens were scanned using a Personal Molecular Imager (Bio-Rad), and band densities were quantified using ImageJ software (52).

For *in vitro* Abl2 activation experiments, 1 μM purified recombinant Abl2 constructs were preincubated with 10 nM PDGFRβ CD for 2 h at 32 °C in 25 mM Hepes at pH 7.25, 150 mM NaCl, 5% glycerol, 5 mM MgCl_2_, 5 mM MnCl_2_, 1 mM sodium pervanadate, 1 mM DTT, and 10 μM cold ATP. After 1 h of preincubation in room temperature, 25-μl reactions were initiated by addition of GST-CrkII (0–16 μM as substrate), 1 nM of preincubated Abl2 kinase proteins, 5 μM ATP, and 0.5 μCi of [γ-^32^P] ATP. All reactions were quenched with 1× LSB after 10 min, boiled, and separated on 10% SDS-PAGE gels. Gels were stained with Blue Silver G-250 Coomassie for 30 min to visualize GST-CrkII protein bands. Bands were cut out, along with background regions within the same lane, and scintillation counted along with a 1 μl sample from the kinase assay. The number of counts per minute was calculated, and *K*_*M*_ and *k*_cat_ values were determined as previously (9, 26).

### Cell culture, construct transfection, and antibodies

Experiments were performed in HEK293 cells (American Type Culture Collection) and *mycoplasma*-free WT mouse 3T3 fibroblast cells. Cells were grown in Dulbecco's modified Eagle's medium supplemented with 10% fetal bovine serum, 100 units/ml penicillin, 100 μg/ml streptomycin, and 2 mM l-glutamine. *abl2*^−/−^ and *abl1*^*−/−*^abl2^*−/−*^ 3T3 fibroblasts were generated using CRISPR/Cas9. A guide sequence of 5′-CATGTAAAGTAACACGACGG-3′ with a protospacer adjacent motif (CGG) targeting the seventh exon of Abl2 was inserted into lentiCRISPRv2 plasmid (Addgene Plasmid #52961), then transfected into HEK293T cells to generate Abl2sg1 lentivirus. WT mouse 3T3 fibroblast cells were infected with the generated Abl2sg1 lentivirus and then selected with 2 μg/ml puromycin for 72 h to generate *abl2*^*−/−*^ 3T3 cells. Another guide sequence of 5′-GTTAGTTCACCATCACTCCA-3′ with a protospacer adjacent motif (CGG) targeting the fourth exon of Abl1 was inserted into lentiCRISPRv2 neo plasmid (Addgene Plasmid #98292), then transfected into HEK293T cells to generate Abl1sg1 lentivirus. WT mouse 3T3 fibroblast cells were infected with the generated Abl2sg1 lentivirus and Abl1sg1 lentivirus simultaneously and then selected with 2 μg/ml puromycin and 800 μg/ml G418 to generate *abl1*^*−/−*^*abl2*^*−/−*^ DKO 3T3 cells.

The following antibodies were used for this study: phosphotyrosine (4G10; Upstate/Millipore or affinity purified from hybridomas), (P)-Y751 PDGFRβ (Cell Signaling), CrkII (Cell Signaling), (P)-Y439 Abl2 (Thermo Fisher Scientific), (P)-Y221 CrkII (Cell Signaling), Abl2 (Ar11, Ar19; purified from hybridomas), HA (12CA5 purified from hybridomas), PR4, a rabbit polyclonal antiserum recognizing the C-terminal 13 amino acids of the human PDGFRβ was a generous gift from Daniel Dimaio (Yale University).

### *In vivo* PDGFRβ pulldown binding assay

HEK293 cells were transiently transfected with WT or mutant full-length PDGFRβ using polyethylenimine transfection. At 48 h after transfection, cells were serum starved overnight with Dulbecco's modified Eagle's-only medium. PDGFRβ was then stimulated with 100 nM of PDGF-BB for 10 min. Cells were lysed in radioimmunoprecipitation assay buffer (50 mM Hepes at pH 7.25, 150 mM NaCl, 1% Nonidet P-40, 1 mM EDTA, 1% deoxycholic acid, 0.1% SDS, 0.5 mM sodium pervanadate, and protease inhibitor). Lysates (500 μg) were incubated with 1 μM of Abl2 SH2-linked beads in a 500-μl reaction overnight before washing and resuspending in LSB. Pulldown products were boiled, separated by SDS-PAGE, and then immunoblotted for PDGFRβ.

### Immunoprecipitation

Abl2-HA was immunoprecipitated from HEK298 cells lysed in Triton lysis buffer (25 mM Hepes at pH 7.25, 150 mM NaCl, 1 mM EDTA,10% glycerol, 1% Triton X-100, 0.5 mM sodium pervanadate, and protease inhibitor). Cell lysate (0.5 mg; standardized to 1 mg/ml) was precleared with 20 μl of Protein A/G Plus Agarose bead (Thermo Fisher Scientific) for 1 h at 4 °C. The precleared supernatant was incubated with 20 μl of beads that had been incubated overnight with anti-HA antibody (12CA5) for 1 h at 4 °C. Immunoprecipitates were washed three times with 0.5 ml of lysis buffer, suspended in 40 μl of LSB, and separated by SDS-PAGE for immunoblot analysis.

### Measurement of CrkII phosphorylation

WT and *abl1*^*−/−*^*abl2*^*−/−*^ DKO KO mouse 3T3 fibroblasts were serum starved overnight before stimulation with 100 nM of PDGF-BB for 10 min. Cells were lysed in radioimmunoprecipitation assay buffer (50 mM Hepes at pH 7.25, 150 mM NaCl, 1% Nonidet P-40, 1 mM EDTA, 1% deoxycholic acid, 0.1% SDS, 0.5 mM sodium pervanadate, and protease inhibitor). Lysate (40 μg) in LSB was boiled, separated by SDS-PAGE, and then blotted for CrkII, (P)-Y221 CrkII, PDGFRβ, and (P)-Y751 PDGFRβ. About 500 μg of cell lysate was precleared with A/G-agarose beads (Pierce Protein Biology) and incubated overnight at 4 °C with Ar11 antibody beads to immunoprecipitate Abl2. Immunocomplexes were incubated with protein A/G-agarose beads for 1 h at 4 °C before spinning down, washing, and resuspending in LSB. Pulldown products were boiled, separated by SDS-PAGE, transferred, and then immunoblotted for Abl2 and phosphotyrosine.

### In solution proteolysis and phosphopeptide enrichment

*In vitro* phosphorylated Abl2 (50 mg) was reduced with DTT (10 mM, 30 min, 56 °C), alkylated with iodoacetamide (30 mM, room temperature, 45 min in dark), and then digested with trypsin in a trypsin-to-protein ratio of 1:20 at 37 °C overnight. The reaction was quenched by 1% formic acid (FA; final concentration). The resulting peptides were dried using a SpeedVac (Thermo; SPD1010), desalted with Pierce C18 tips (Thermo), and the phosphopeptides were enriched by Titansphere Phos-TiO Kit (GL Science) according to the manufacturer's protocol.

### LC–MS/MS analysis

The enriched phosphopeptides were analyzed by MS using a Dionex Ultimate 3000 nano-UHPLC system LC coupled with a Thermo Scientific Orbitrap Velos Pro mass spectrometer. The Dionex Ultimate 3000 system was equipped with an Acclaim PepMap 100 (C18, 5 μm, 100 Å, 100 μm × 2 cm; Thermo Fisher Scientific) trap column and an Acclaim PepMap RSLC (C18, 2 μm, 100 Å, 75 μm × 50 cm; Thermo Scientific) analytical column. Chromatographic separation of the phosphopeptides was achieved using a linear gradient consisted of ultrapure water (J. T. Baker; Thermo Fisher Scientific) with 0.1% FA and acetonitrile (J. T. Baker; Thermo Fisher Scientific) with 0.1% FA, where the gradient was from 5% B at 0 min to 40% B at 105 min. The source voltage was set in 2.1 kV, and the capillary temperature was set at 320 °C. The MS analysis was performed using a top-10 data-dependent analysis in the positive ion mode with dynamic exclusion option enabled for 30 s. MS/MS spectra were collected using collision-induced dissociation. Data were searched against a custom-made database, which included the sequence of recombinant Abl2 in the background of *E. coli* database (UniProtKB; February 2018, 4435 annotated entries) using the Sequest HT algorithm. The Proteome Discoverer, version 2.5 (Thermo Fisher Scientific) was used for database search with the following parameters: enzyme, trypsin (full); parent mass error tolerance, 10 ppm; fragment mass error tolerance, 0.6 Da (monoisotopic); maximum number of missed cleavage sites, two; variable modifications of +15.995 Da (oxidation) on methionine, and +79.996 Da (phosphorylation) on serine, threonine, and tyrosine; fixed modification of +57.021 Da (carbamidomethylation) on cysteine. Identified peptides were validated through the false discovery rate, for which thresholds were 0.01 and 0.05 for strict and relaxed target false discovery rate, respectively.

## Data availability

All data are contained within the article and the supporting information. The MS data have been deposited to the ProteomeXchange Consortium *via* the PRIDE (53) partner repository with the dataset identifier PXD025896 and 10.6019/PXD025896.

## Supporting information

This article contains supporting information.

## Conflict of interest

The authors declare that they have no conflicts of interest with the contents of this article.
