# Supplementary material for: Platelet-derived growth factor receptor beta activates Abl2 via direct binding and phosphorylation
Source: J Biol Chem. 2021 Jun 16;297(1):100883. doi: 10.1016/j.jbc.2021.100883 (PMC8259415; doi:10.1016/j.jbc.2021.100883)
Supplement: Figure S1 [file mmc1.pdf]

Supplemental Figure S1

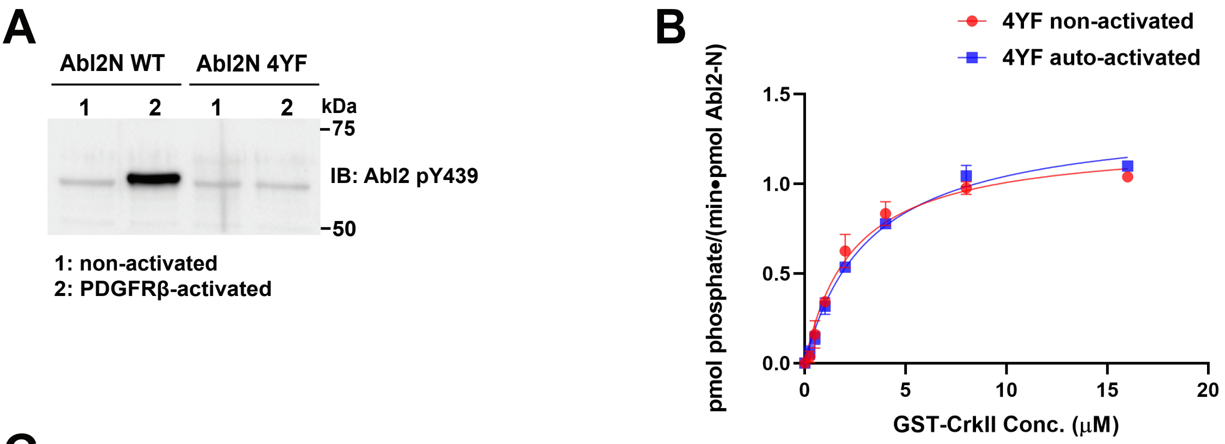

C

Kinase	Preincubation condition	K_M (μM)	k_{cat} (min^{-1})	k_{cat} / K_M ($min^{-1}μM^{-1}$)	$Δk_{cat}$ / K_M ($min^{-1}μM^{-1}$)
Abl2N 4YF	non-activated	2.34 ± 0.61	1.24 ± 0.11	0.53	1.00
Abl2N 4YF	auto-activated	3.19 ± 0.59	1.37 ± 0.09	0.43	0.83

Figure S1: (A) Abl2-N 4YF (Y116F, Y161F, Y272F, Y310F) was incubated with PDGFRβ in an activation reaction. 5 μg of reaction product were immunoblotted with Abl2 pY439 antibody. (B) Kinase activity was assayed by determining the kinetic parameter of GST-CrkII phosphorylation in [γ-32P] ATP kinase assays. Measurements collected along an increasing concentration (0-16 μM) of CrkII in each condition were fit to Michaelis-Menten isotherms using GraphPad. Error bars represent the S.E. from n = 3 concentration series for each condition. (C) KM, kcat and the catalytic efficiency (kcat/KM) values of Abl2-N 4YF mediated GST-CrkII phosphorylation were calculated from isotherms fit to perspective conditions shown in B.
