# Supplementary material for: Platelet-derived growth factor receptor beta activates Abl2 via direct binding and phosphorylation
Source: J Biol Chem. 2021 Jun 16;297(1):100883. doi: 10.1016/j.jbc.2021.100883 (PMC8259415; doi:10.1016/j.jbc.2021.100883)

MS/MS spectra for all phosphopeptide

Y139

Sequence: VLGYNQNGEWSEVR, Y4-Phospho (79.96633 Da)

Charge: +2, Monoisotopic m/z: 865.88333 Da (+3 mmu/+3.46 ppm), MH+: 1730.75938 Da, RT: 41.7746 min,

Identified with: Sequest HT (v1.17); XCorr:4.12, Percolator q-Value:0.0e0, Percolator PEP:4.4e-4, ptmRS: Best Site Probabilities:Y4(Phospho): 100, Ions matched by search engine: 0/0

Fragment match tolerance used for search: 0.6 Da

Fragments used for search: b; b-H_2O; b-NH_3; y; y-H_2O; y-NH_3

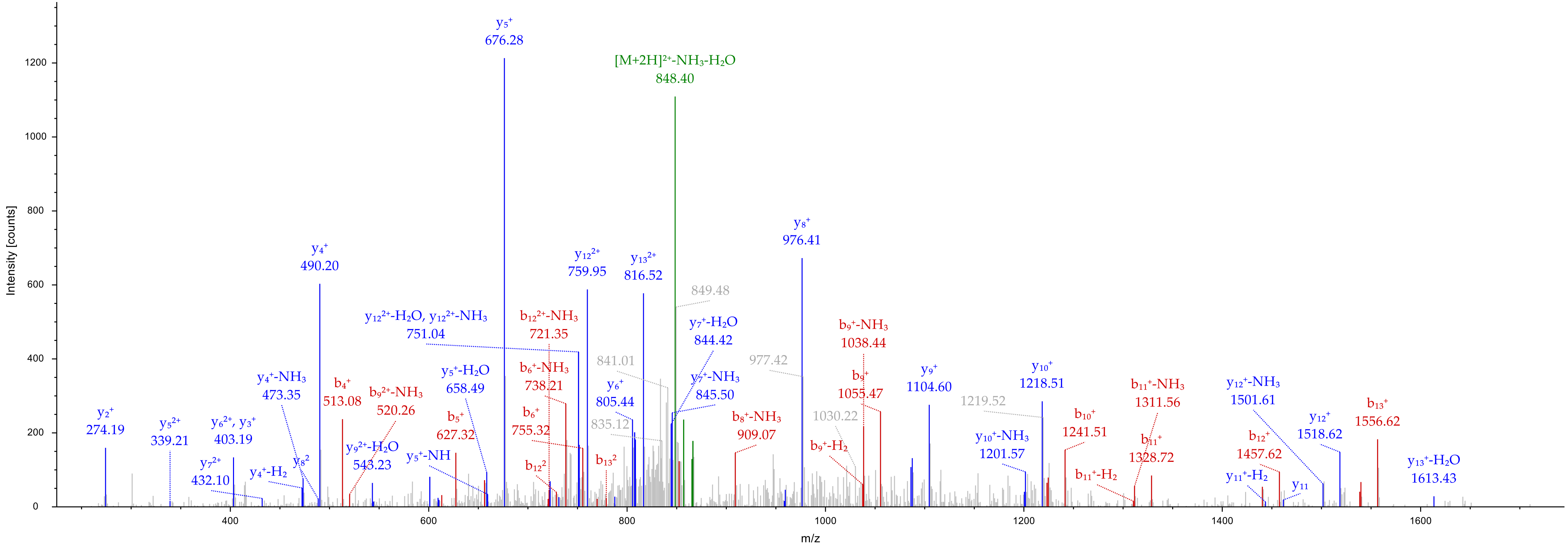

Y161

Sequence: NGQGWVPSNYITPVNSLEK, Y10-Phospho (79.96633 Da)

Charge: +2,   Monoisotopic m/z: 1092.00988 Da (-2.2 mmu/-2.01 ppm),   MH+: 2183.01248 Da,   RT: 60.2746 min,

Identified with: Sequest HT (v1.17); XCorr:3.65, Percolator q-Value:0.0e0, Percolator PEP:1.8e-3, ptmRS: Best Site Probabilities:Y10(Phospho): 100, Ions matched by search engine: 0/0

Fragment match tolerance used for search: 0.6 Da

Fragments used for search: b; b-H_2O; b-NH_3; y; y-H_2O; y-NH_3

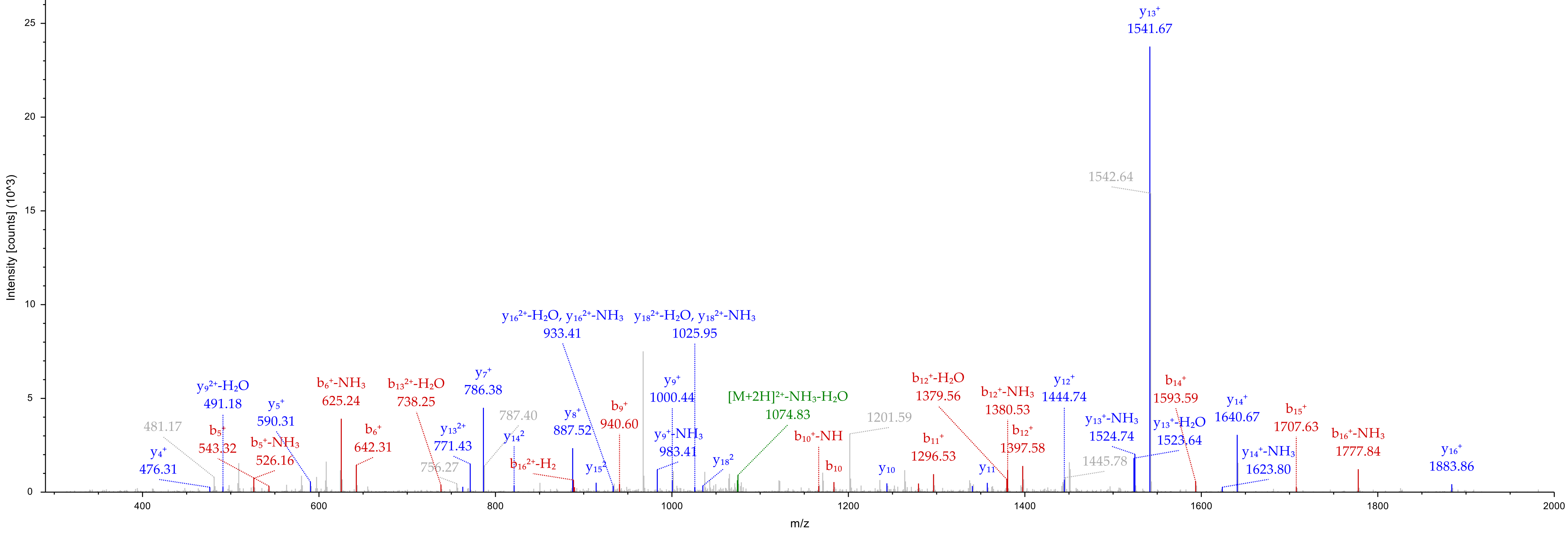

Y272

Sequence: CNKPTVYGVSPIHDK, C1-Carbamidomethyl (57.02146 Da), Y7-Phospho (79.96633 Da)
Charge: +3, Monoisotopic m/z: 598.94623 Da (-0.1 mmu/-0.17 ppm), MH+: 1794.82414 Da, RT: 17.4945 min,
Identified with: Sequest HT (v1.17); XCorr:3.77, Percolator q-Value:0.0e0, Percolator PEP:4.3e-3, ptmRS: Best Site Probabilities:Y7(Phospho): 100, Ions matched by search engine: 0/0
Fragment match tolerance used for search: 0.6 Da
Fragments used for search: b; b-H_2O; b-NH_3; y; y-H_2O; y-NH_3

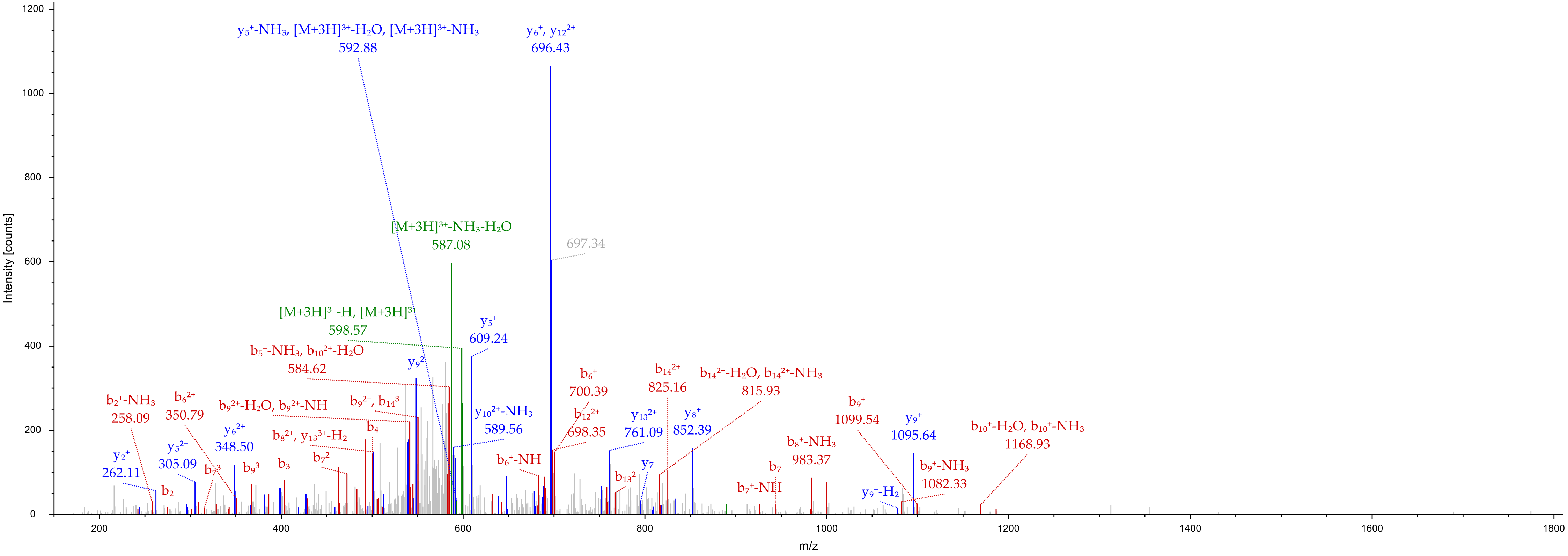

Y299

Sequence: LGGGQYGEVYVGVWK, Y6-Phospho (79.96633 Da)
Charge: +2, Monoisotopic m/z: 846.39596 Da (+0.88 mmu/+1.03 ppm), MH+: 1691.78465 Da, RT: 58.7613 min,
Identified with: Sequest HT (v1.17); XCorr:3.66, Percolator q-Value:0.0e0, Percolator PEP:6.7e-6, ptmRS: Best Site Probabilities:Y6(Phospho): 100, Ions matched by search engine: 0/0
Fragment match tolerance used for search: 0.6 Da
Fragments used for search: b; b-H_2O; b-NH_3; y; y-H_2O; y-NH_3

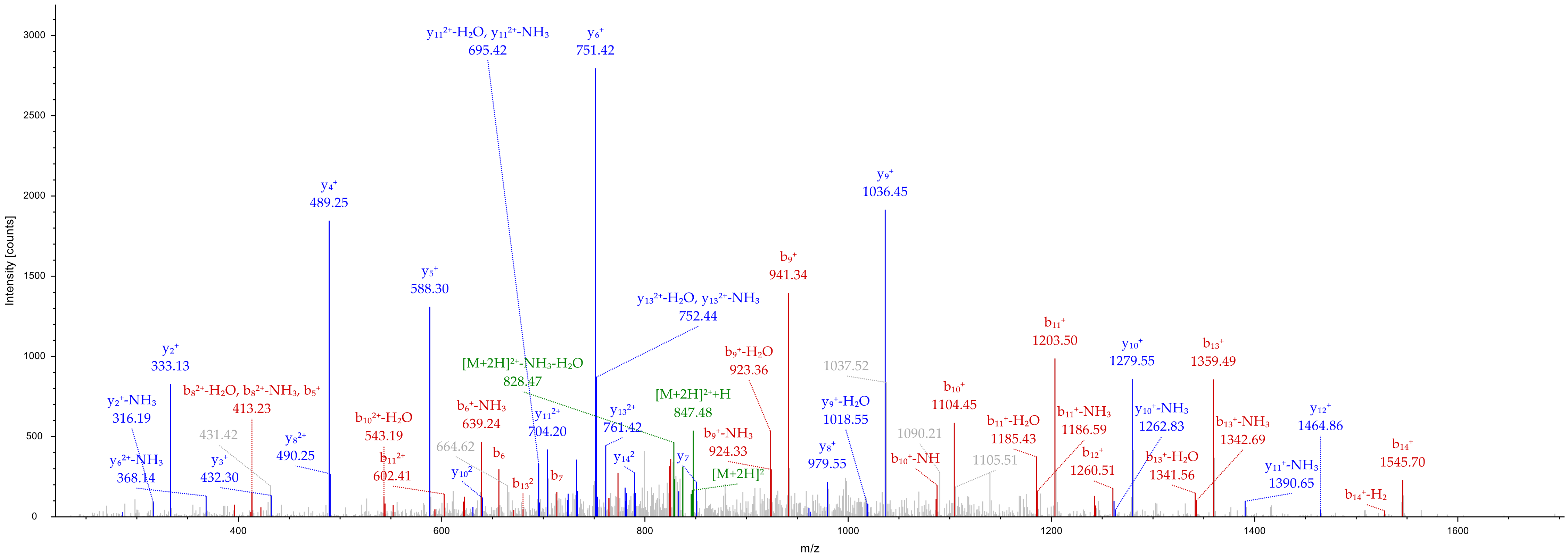

Y303

Sequence: LGGGQYGEVYVGVWK, Y10-Phospho (79.96633 Da)
Charge: +2, Monoisotopic m/z: 846.39511 Da (+0.02 mmu/+0.02 ppm), MH+: 1691.78294 Da, RT: 59.3489 min,
Identified with: Sequest HT (v1.17); XCorr:4.11, Percolator q-Value:0.0e0, Percolator PEP:4.2e-3, ptmRS: Best Site Probabilities:Y10(Phospho): 100, Ions matched by search engine: 0/0
Fragment match tolerance used for search: 0.6 Da
Fragments used for search: b; b-H_2O; b-NH_3; y; y-H_2O; y-NH_3

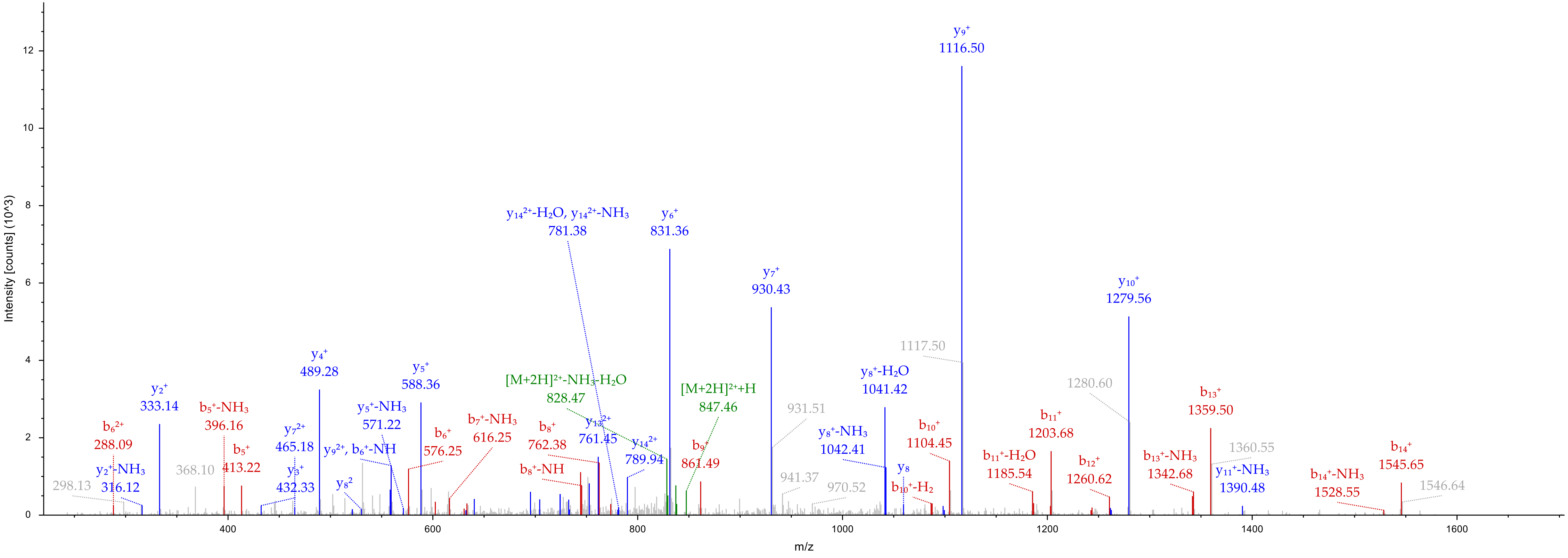

Y310

Sequence: KYSLTVAVMTLK, Y2-Phospho (79.96633 Da), M9-Oxidation (15.99492 Da)
Charge: +3,   Monoisotopic m/z: 483.91893 Da (-0.01 mmu/-0.02 ppm),   MH+: 1449.74225 Da,   RT: 45.8636 min,
Identified with: Sequest HT (v1.17); XCorr:3.42, Percolator q-Value:0.0e0, Percolator PEP:2.0e-3, ptmRS: Best Site Probabilities:Y2(Phospho): 100,
Ions matched by search engine: 0/0
Fragment match tolerance used for search: 0.6 Da
Fragments used for search: b; b-H_2O; b-NH_3; y; y-H_2O; y-NH_3

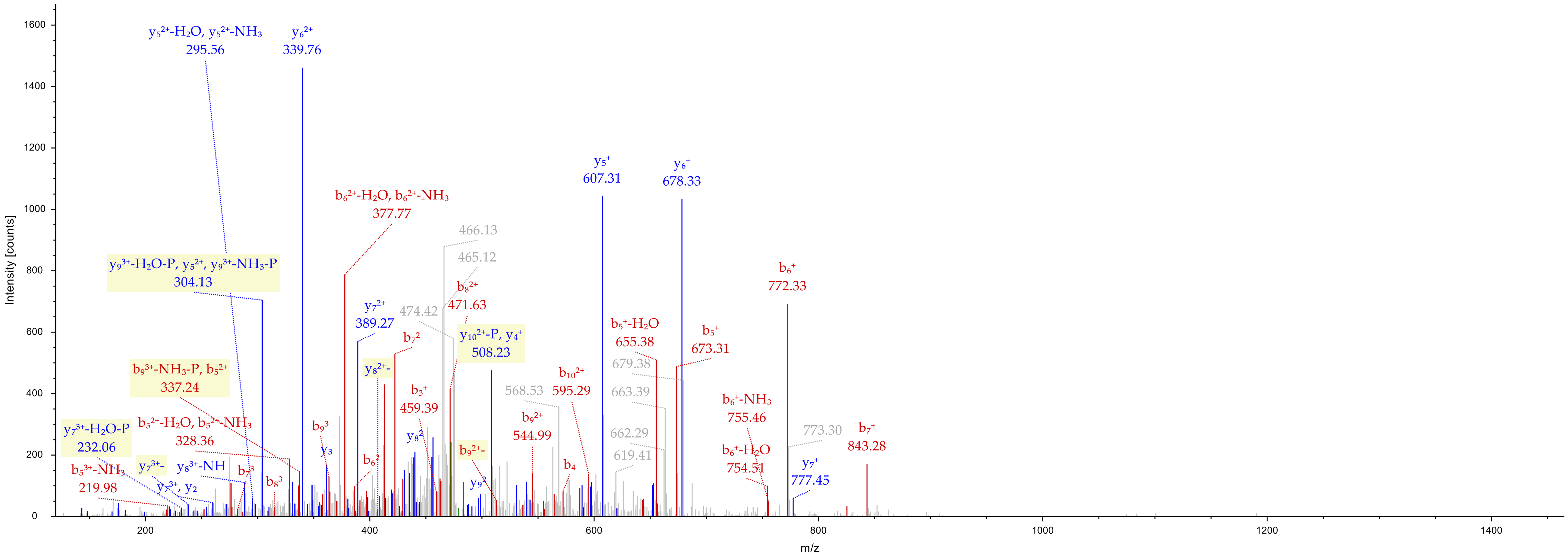

Y439

Sequence: LMTGDTYTAHAGAK, Y7-Phospho (79.96633 Da), M2-Oxidation (15.99492 Da)
Charge: +2, Monoisotopic m/z: 766.82528 Da (-0.91 mmu/-1.18 ppm), MH+: 1532.64327 Da, RT: 18.3106 min,
Identified with: Sequest HT (v1.17); XCorr:2.28, Percolator q-Value:3.0e-3, Percolator PEP:8.7e-2, ptmRS: Best Site Probabilities:Y7(Phospho): 100, Ions matched by search engine: 0/0
Fragment match tolerance used for search: 0.6 Da
Fragments used for search: b; b-H_2O; y; y-H_2O; y-NH_3

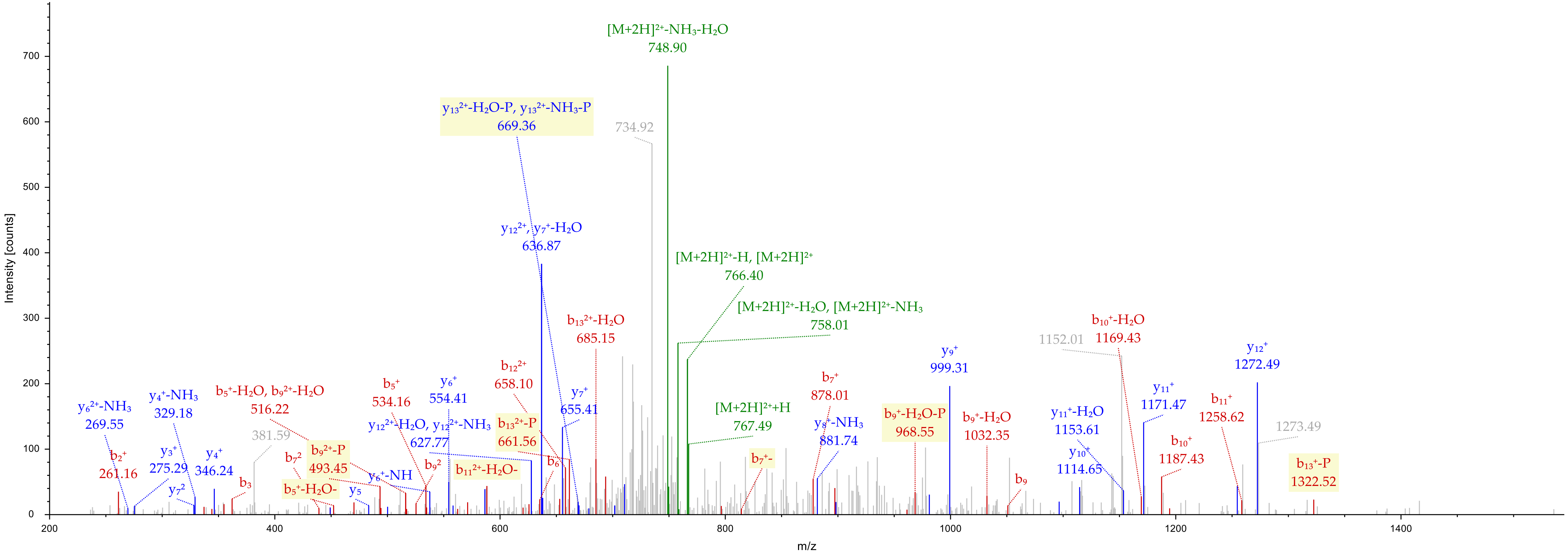

Supplement: Supporting information [file mmc2.pdf]
